# Association of Retinal Blood Flow with Progression of Visual Field in Glaucoma

**DOI:** 10.1038/s41598-019-53354-4

**Published:** 2019-11-14

**Authors:** Soo Ji Jeon, Da-Young Shin, Hae-Young Lopilly Park, Chan Kee Park

**Affiliations:** Department of Ophthalmology, Seoul St. Mary’s Hospital, College of Medicine, The Catholic University of Korea, Seoul, Republic of Korea

**Keywords:** Risk factors, Outcomes research

## Abstract

In the glaucoma clinic, patients with normal intraocular pressure (IOP) can sometimes show visual field (VF) progression. Therefore, clarification of relationship between vascular status and glaucomatous VF deterioration is a focus of interest. We used optical coherence tomography angiography (OCTA), with the aim of evaluating the relationship between vessel density (VD) and VF progression in glaucoma patients. We included 104 eyes with open angle glaucoma who were followed up for at least 5 years in this retrospective case-control study. Superficial and deep VD of macula were assessed by OCTA. Regression analysis and Cox proportional hazards model were used to identify factors significantly associated with VF progression. In logistic regression analysis determining VF progression from Guided Progression Analysis (GPA) program, initial IOP and deep macular VD were significantly associated with VF progression in multivariate analysis (*P* = 0.019 and 0.004). Cox proportional hazards model also identified deep macular VD as significantly related to VF progression (P = 0.035). In conclusion, initial IOP and deep VD were related to VF deterioration in glaucoma. Deep VD might be used as a surrogate of glaucomatous VF progression related with vascular incompetence.

## Introduction

Glaucoma is a progressive disease that involves thinning of the retinal nerve fiber layer (RNFL) and defects in the corresponding areas of the visual field (VF). Uncontrolled intraocular pressure (IOP) has traditionally been considered to be the only modifiable risk factor associated with progression in glaucoma, with the reduction of IOP as the mainstay of treatment^1,2^. However, there are patients who still show glaucomatous visual field progression even with well controlled IOP^3^. This indicates that there must be additional factors involved in the progression of glaucoma.

According to the Collaborative Normal-Tension Glaucoma Study (CNTG) and the Early Manifest Glaucoma Trial (EMGT), migraine and disc hemorrhage contributed to the progression of visual field deterioration apart from IOP^4,5^. Several other studies have also shown that decreased retrobulbar blood flow or impaired systemic circulation was associated with glaucoma progression^6,7^.

Recently, optical coherence tomography angiography (OCTA) has been used taking the advantage of noninvasiveness and rapid imaging. As an emerging technology that could visualize vascular circulation without dye injection, this machine has become an additional tool for clinical diagnosis and treatment at a rapid rate. For glaucoma specialist, OCTA has major advantages that could afford direct and layer-specific imaging of the circulation of blood in the macula and peripapillary region. Several studies have utilized OCTA to study the relationships between decreased vessel density (VD), incidence of glaucoma, and severity of visual field defects^8–10^. However, whether low VD is a cause of glaucomatous damage or is merely an epiphenomenon of retinal thinning remains difficult to clarify. The deep retinal microvasculature might be less affected by structural changes due to the occurrence of retinal thinning mainly in the superficial retinal nerve fiber layer (RNFL) and ganglion cell layer (GCL) in glaucoma. A previous study found that deep VD located in the the inner nuclear layer (INL) was an independent risk factor of central visual function in addition to retinal thickness thinning, and it was suggested that deep VD assessed by OCTA might be a biomarker for ocular blood flow^11^.

The purpose of this study was to evaluate the relationship between retinal VD and glaucomatous VF progression, and to identify an indicator of ocular vascular stress that is associated with VF deterioration.

## Results

A total of 151 eyes of patients with open angle glaucoma (OAG) were involved in this study. Five eyes were excluded because of repeated unreliable VF test results, as were 2 eyes of patients who exhibited poor compliance with IOP lowering treatment. In addition, 40 eyes showing inadequate OCTA image quality were excluded, leaving a total of 104 eyes.

Table 1 shows the baseline characteristics of the study participants. All patients had normal IOPs when they were diagnosed with glaucoma. The mean follow-up duration was 110.30 ± 42.76 months, and the SITA 24-2 MD was −4.44 ± 5.68 dB, consistent with mild glaucomatous defect.

**Table 1:** Demographic and ocular characteristics of study subjects.

	Total n = 104
Age, years	58.21 (±13.11)
Sex, male:female	42:62
Hypertension, n(%)	34 (32.7%)
Diabetes, n(%)	8 (7.7%)
Disc hemorrhage, n(%)	7 (6.7%)
Follow up duration, months	110.30 (±42.76)
Axial length, mm	24.87 (±1.38)
Best corrected visual acuity, decimal	0.90 (±0.12)
Initial intraocular pressure, mmHg	15.28 (±3.26)
Average intraocular pressure, mmHg	13.64 (±2.81)
IOP fluctuation (SD of IOP)	1.37 (±0.78)
Initial RNFL average thickness, μm	73.35 (±11.89)
Last RNFL average thickness, μm	69.38 (±12.45)
Initial SITA 24-2 MD, dB	−4.44 (±5.68)
Initial SITA 24-2 PSD, dB	5.08 (±4.35)
Last SITA 24-2 MD, dB	−5.83 (±6.46)
Last SITA 24-2 PSD, dB	6.21 (±4.48)
Superficial VD, %	24.70 (±3.45)
Deep VD, %	31.01 (±2.13)

IOP: intraocular pressure; MD: mean deviation; PSD: pattern standard deviation; VD: vessel density; RNFL: retinal nerve fiber layer; PPA: peripapillary area.

Data are means (±SD) or numbers (%), as appropriate.

The rate of change in the VF was determined by calculating the MD slope of the VF. Table 2 presents the correlation coefficients relating to the MD slope with clinical or ocular parameters, including OCT parameters and VDs derived from OCTA images. Deep VD determined by OCTA was marginally correlated with MD slope (*P* = 0.058). According to linear regression analysis, the association of deep VD with the MD slope of VF was significant, with the regression line showing a positive gradient (ß = 0.046 and *P* = 0.018 by multivariate analysis). In other words, the higher the VD, the lower the deterioration rate in the VF. The initial IOP of each study participant was also marginally significant by multivariate analysis (*P* = 0.051) (Table 3).

**Table 2:** Correlation coefficients between the MD slope and clinical or ocular parameters.

	r	R^2^	*P* value
Age	0.047	0.002	0.633
Follow up duration	−0.049	0.002	0.620
Initial IOP	−0.150	0.022	0.129
Average IOP	−0.023	0.001	0.909
IOP fluctuation	−0.012	0.000	0.950
Initial MD of SITA 24-2	0.047	0.002	0.634
**OCT parameters**			
RNFL thickness	−0.005	0.000	0.959
Average CDR	−0.023	0.001	0.827
Rim area	0.116	0.014	0.277
Disc area	0.094	0.009	0.380
Cup volume	0.037	0.001	0.733
RNFL slope*	0.073	0.005	0.552
**OCT angiography**			
Superficial VD	0.125	0.016	0.208
Deep VD	0.187	0.035	0.058

IOP: intraocular pressure; MD: mean deviation; OCT: optical coherence tomography; RNFL: retinal nerve fiber layer; CDR: cup-disc ratio; VD: vessel density.

*RNFL slope (μm/yr) = (Change in mean RNFL thickness during follow-up period) / (follow-up duration).

Pearson correlation analysis was used.

**Table 3:** Univariate and multivariate regression analysis of the MD slope in glaucoma patients.

	Univariate			Multivariate		
	ß	95% CI	*P* value	ß	95% CI	*P* value
Age	0.002	−0.007 to 0.011	0.633			
Follow up duration	0.001	−0.002 to 0.001	0.620			
Initial MD of SITA 24-2	0.003	−0.009 to 0.015	0.634			
Initial IOP	−0.016	−0.037 to 0.005	0.129	−0.024	−0.048 to 0.000	0.051
Average IOP	−0.026	−0.077 to 0.025	0.307			
IOP fluctuation	−0.006	−0.195 to 0.184	0.950			
RNFL thickness	0.000	−0.006 to 0.006	0.959			
Average CDR	−0.099	−0.998 to 0.800	0.827			
Rim area	0.214	−0.175 to 0.603	0.277	0.274	−0.152 to 0.700	0.204
Disc area	0.088	−0.110 to 0.287	0.380			
Cup volume	0.054	−0.257 to 0.365	0.733			
RNFL slope	0.017	−0.039 to 0.073	0.552			
Superficial VD	0.013	−0.007 to 0.033	0.208	−0.003	−0.027 to 0.022	0.836
Deep VD	0.031	−0.001 to 0.063	0.058	0.046	0.008 to 0.084	0.018

CI: confidence interval; MD: mean deviation; IOP: intraocular pressure; RNFL: retinal nerve fiber layer; CDR: cup-disc ratio; VD: vessel density

Multivariate regression analysis included factors of *P* values lower than 0.3 in univariate analysis.

According to guided progression analysis (GPA) program, 55 eyes showed significant progression of VF loss during the follow-up period, while 49 eyes had not progressed. As shown in Table 4, the differences in age, gender, frequency of disc hemorrhage, axial length, visual acuity, and RNFL thickness between the progressors and nonprogressors were not significant. The initial MD values of SITA 24-2 were comparable between the two group, however, the final MD and MD slope were significantly worse for the progressors (−7.38 ± 6.75 dB of last MD and −0.34 ± 0.35 dB/yr of MD slope in progressor; −4.08 ± 5.69 dB of last MD and −0.02 ± 0.27 dB/yr of MD slope in non-progressor; all *P* < 0.001). As expected, the RNFL change slope was steeper (−1.05 ± 1.05 μm/yr) and initial IOP was higher (16.08 ± 3.21 mmHg) in the progressor group compared to non-progressor group (−0.16 ± 1.411 μm/yr and 14.81 ± 3.17 mmHg, *P* = 0.010 and 0.017, respectively). Interestingly, deep VD was significantly lower in the progressor group (30.40 ± 2.11% in progressor and 31.68 ± 1.96% in non-progressor; *P* = 0.002).

**Table 4:** Comparisons between glaucoma patients with or without visual field progression.

	Progressor (n = 55)	Non-progressor (n = 49)	*P* value
Age, years	58.91 (±12.21)	57.43 (±14.21)	0.500
Sex, male:female	24:31	18:31	0.474
Hypertension, n(%)	18 (32.7%)	16 (32.7%)	0.994
Diabetes, n(%)	5 (10.2%)	3 (5.5%)	0.364
Disc hemorrhage, n(%)	5 (9.1%)	2 (4.1%)	0.309
Axial length, mm	24.78 (±1.51)	24.99 (±1.20)	0.583
Best corrected visual acuity, decimal	0.91 (±0.13)	0.89 (±0.12)	0.366
Initial IOP, mmHg	16.08 (±3.21)	14.81 (±3.17)	0.017
Average IOP, mmHg	14.35 (±3.04)	12.64 (±2.24)	0.120
IOP fluctuation (SD of IOP)	1.42 (±0.85)	1.30 (±0.68)	0.691
Initial RNFL average thickness, μm	73.69 (±12.47)	72.96 (±11.32)	0.756
Last RNFL average thickness, μm	68.04 (±13.01)	70.88 (±11.74)	0.247
RNFL slope, μm/yr	−1.05 (±1.05)	−0.16 (±1.41)	0.010
Initial SITA 24-2 MD, dB	−4.81 (±5.83)	−4.01 (±5.54)	0.476
Initial SITA 24-2 PSD, dB	5.37 (±4.37)	4.75 (±4.35)	0.470
Last SITA 24-2 MD, dB	−7.38 (±6.75)	−4.08 (±5.69)	0.009
Last SITA 24-2 PSD, dB	7.48 (±4.29)	4.77 (±4.29)	0.002
MD slope, dB/year	−0.34 (±0.35)	−0.02 (±0.27)	<0.001
PSD slope, dB/year	0.30 (±0.39)	−0.01 (±0.22)	<0.001
Superficial VD, %	24.62 (±3.58)	24.78 (±3.32)	0.810
Deep VD, %	30.40 (±2.11)	31.68 (±1.96)	0.002

IOP: intraocular pressure; SD: standard deviation; RNFL: retinal nerve fiber layer; MD: mean deviation; PSD: pattern standard deviation; VD: vessel density.

Data are means (±SD) or numbers (%), as appropriate.

Student’s t test and Chi-square test were used.

Table 5 shows the results of logistic regression analysis, which was used to identify those factors that were related to the glaucomatous progression of VF loss as determined by GPA. Follow-up duration, initial IOP, cup volume from initial OCT and deep VD from OCTA showed significant or marginal significance by univariate analysis (*P* values ranging from 0.003 to 0.106). By multivariate analysis, initial IOP and deep VD were significantly associated with VF progression (*P* = 0.019 and 0.004, respectively).

**Table 5:** Logistic regression analysis of visual field progression in glaucoma patients.

	Univariate			Multivariate		
	Exp(ß)	95% CI	*P* value	Exp(ß)	95% CI	*P* value
Age	0.985	0.938 to 1.035	0.554			
Follow up duration	1.008	0.998 to 1.018	0.106	1.009	0.992 to 1.026	0.318
Initial IOP	1.110	0.981 to 1.256	0.099	1.575	1.131 to 2.195	0.019
Average IOP	0.084	0.815 to 1.451	0.570			
IOP fluctuation	1.235	0.456 to 3.347	0.678			
Initial MD of SITA 24-2	0.975	0.909 to 1.045	0.473			
RNFL thickness	1.005	0.973 to 1.039	0.753			
Average CDR	15.864	0.118 to 2136.641	0.269			
Rim area	0.815	0.099 to 6.697	0.849			
Disc area	2.091	0.690 to 6.334	0.192	4.186	0.295 to 59.378	0.290
Cup volume	4.357	0.742 to 25.589	0.103	0.125	0.002 to 8.957	0.340
Superficial VD	0.986	0.881 to 1.103	0.807			
Deep VD	0.735	0.600 to 0.901	0.003	0.548	0.374 to 0.803	0.004

CI: confidence interval; MD: mean deviation; IOP: intraocular pressure; RNFL: retinal nerve fiber layer; CDR: cup-disc ratio; VD: vessel density

Multivariate regression analysis included factors of *P* values lower than 0.2 in univariate analysis.

Considering the varied follow-up periods of participants and the marginal significance of the association of follow-up period with VF progression by logistic analysis, we used Cox proportional-hazards models to identify meaningful factors affecting VF deterioration. By univariate analysis, age at last follow-up and deep VD had marginal significance (*P* = 0.082 and 0.085, respectively), and multivariate analysis showed that the previous two factors were significantly associated with VF progression (*P* = 0.005 and 0.007, respectively, Table 6). The hazard ratio of deep VD was 0.548, indicating that a higher VD value was less related to VF progression (Table 6).

**Table 6:** Risk factors affecting visual field progression using Cox proportional-hazards models.

	Univariate			Multivariate		
	HR	95% CI	*P* value	HR	95% CI	*P* value
Age	0.969	0.947 to 0.992	0.082	1.541	1.138 to 2.088	0.005
Initial IOP	1.070	0.982 to 1.166	0.122	1.261	0.978 to 1.627	0.073
Average IOP	1.128	0.967 to 1.316	0.125			
IOP fluctuation	1.657	0.824 to 3.332	0.156			
Initial MD of SITA 24-2	1.000	0.958 to 1.044	0.987			
RNFL thickness	1.007	0.984 to 1.030	0.555			
Average CDR	0.798	0.026 to 24.205	0.897			
Rim area	1.063	0.264 to 4.287	0.931			
Disc area	0.870	0.392 to 1.929	0.731			
Cup volume	1.011	0.348 to 2.933	0.985			
Superficial VD	1.037	0.950 to 1.132	0.421	1.308	0.975 to 1.753	0.073
Deep VD	0.886	0.772 to 1.017	0.085	0.586	0.396 to 0.865	0.007

CI: confidence interval; MD: mean deviation; IOP: intraocular pressure; RNFL: retinal nerve fiber layer; CDR: cup-disc ratio; VD: vessel density.

Multivariate analysis used the backward elimination method.

**Figure 1:**
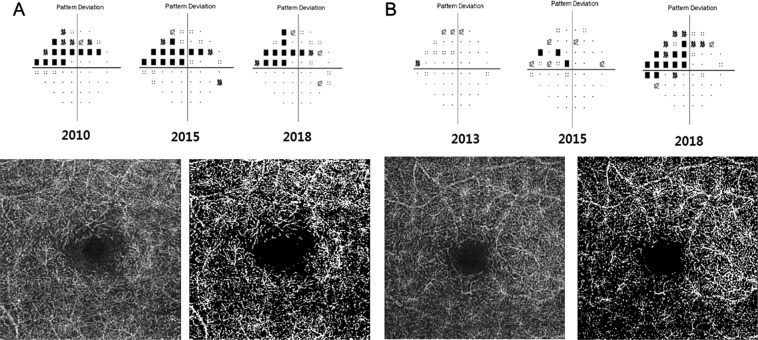
Two representative cases with contrasting features. The lower left image from each case is the original OCT angiography image of deep retinal layer, and the lower right image is the binarized result to calculate vessel density. (**A**) An 81-year-old woman with stable visual field (VF) results; (**B**) a 50-year-old woman with progressed VF. During the follow-up period, case B showed VF progression and lower deep retinal vessel density than that of case A.

Representative cases are shown in Fig. 1. An 81-year-old woman and a 50-year-old woman without any systemic cardiovascular disease had well controlled normal IOP. The 50-year-old woman showed glaucomatous progression of VF loss even with a lower initial MD than that of the 81-year-old woman. The calculated deep VD of the 50-year-old progressor was lower than that of the 81-year-old nonprogressor (31.20% vs 34.02%, respectively).

## Discussion

Ocular blood flow is known to play a critical role in the development and progression of glaucoma^12–14^. However, the direct assessment and identification of ocular blood circulation are difficult. Laser Doppler flowmetry (LDF), laser speckle contrast imaging (LSCI), and magnetic resonance imaging (MRI) have all been used to study ocular blood flow^15^. However, there are several limitations to these approaches, including penetration depth of the laser into ocular tissue^16^, difficulty in quantifying blood flow^17^, the time-consuming nature of the procedures, and the significant cost burden, especially in the outpatient setting. OCTA has come into widespread use in more recent years as a direct indicator of local ocular blood flow. Because OCTA allows higher imaging speeds and obtains volumetric scans that can be segmented by retinal layer to improve image quality and allow for quantitative analysis, it is an important, useful, and practical tool^18,19^.

The vascular plexus of the retinal deep layer is located mainly within INL of retina^20,21^. The INL undergoes little structural change in glaucoma; thus, blood circulation in the INL might help to minimize the effect of resulting decrease of VD due to RNFL and GCL thinning. This point accounts for our continued interest in the deep vascular plexus of the retina. We previously showed that deep VD was not correlated with retinal thickness, even in the INL, where the deep vascular plexus is located^11^. Shin *et al*. focused on the decreased peripapillary VD segmented into superficial and deep retina in glaucoma patients^22^. Lommatzsch *et al*. evaluated macular VD by dividing the retina into superficial and deep layers, and found that the VD in each layer was decreased in glaucoma^23^.

Moghimi *et al*. recently reported that macular VD was related to the rate of RNFL thinning and might be a predictors of the risk of glaucoma progression^24^. However, they considered the vascular plexus of the superficial retina that could be confused with resulting GCL thinning of glaucoma. To strive to overcome these shortcoming, we investigated the VD of deep retinal layer. According to our results, it is suggested that the lower the deep VD, the more progressed VF result. In addition, deep VD was identified as a factor determining the MD slope. During the period of follow-up, the presence of VF deterioration was affected by decreased deep VD, and progressed patients had lower deep VD than non-progressors. Although the causal relationship between these factors is not clear, it is thought that compromised ocular blood circulation underlies VF progression in glaucoma patients with well controlled IOP, and deep VD might reflect the status of the local ocular blood circulation.

In our study, the superficial VD was not associated with VF progression and it is contrasting with the aforementioned study of Moghimi *et al*. However, the OCTA of Moghimi’s study was performed at baseline and ours was performed at different timing. The superficial VD of our study may be affectd by RGC structure of current status – not baseline. According to several studies, glaucoma progression is affected by the baseline state of disease, not by the current state when the patient is treated^25–27^. This difference could make this contradictory results, but the future study with large subjects is required to clarify it.

We had concerns about the effects of topical IOP-lowering medication on retinal VD. Supplementary Table 1 showed the relationships of VDs between different medication groups. Topical prostaglandin did not affect VDs significantly, but patients with topical beta blocker (BB) showed lower superficial VD. The difference might be caused by the different initial IOP and MD for topical BB as shown in Supplementary Table 1. In our subjects, PG eyedrops are often prescribed as the primary choice, and topical BBs tend to be added when trying to strengthen the treatment. The patients with worse glaucoma state or higher IOP were often prescribed the BB and these patients might have thinner GCL thickness – this difference could affect superficial VD. Though, the deep VD located in the INL and not affected by GCL thinning, was not decreased even in the topical BB group. In other words, the deep VD seems not to be significantly affected by topical vasoactive BB.

Our findings incorporated initial IOP as a variable associated with occurrence of VF deterioration. According to several studies including EMGT, higher baseline IOP is consistently found to be a predictive factor for VF progression^28,29^. However, average IOP and IOP fluctuation were not associated with VF progression in this study. Heiji A *et al*^30^. reported that higher mean IOP was associated with more rapid progression, and the study of Matlach^31^ showed the IOP fluctuation as the meaningful factor of glaucoma progression. This controversial results might be due to the different subjects of glaucoma between studies. Our data included examinations from NTG patients but previous studies included mainly POAG patients. In CNTG study, the IOP was not associated with VF progression^32^. Also, in the previous study of our group, mean IOP and IOP fluctuation were not associated with VF progression in NTG patients^33^. However, this controversy should be investigated further with larger study subjects.

There are also many other factors to consider regarding glaucoma progression. We found that when taking into consideration the follow-up period, age was associated with the progression of deterioration in VF. This result in our study was consistent with previous studies asserting that older patients experience greater progression^5,34^. Worse baseline MD was associated with VF progression in several previous studies^25,35^, but this study did not show significant association. Severity of glaucoma in our study subjects was better than −12dB of MD corresponding with mild to moderate glaucoma. Including only patients with limited range of severity could make limitation and a study including with various severity and large number of subjects should be conducted.

Disc hemorrhage is widely known to be related to glaucoma progression^36^. However, in our results, the presence of disc hemorrhage did not show significant relationship with VF progression. There was possibility of undervalued incidence of disc hemorrhage since it can only be confirmed when the patient visits the clinic and this matter could be a limitation of our study.

There are some more limitations in this study. First, OCTA is a recent technology, and obtaining baseline OCTA information on patients is difficult. Patient data collected over the long term is required for evaluating disease progression; therefore, we had to utilize OCT and OCTA data collected from different points in time. However, as we mentioned previously in the discussion, the deep retinal layer is relatively unaffected by glaucoma progression, and under well controlled IOP, there might be minimal compression of blood vessels in the lamina. Thus, deep VD could be used as a surrogate of baseline ocular blood flow. In addition, the retrospective profile of this study could be another limitation. In future study, prospective research that incorporates baseline OCTA data might be more valuable for predicting glaucoma progression. A second limitation of our study was that VF test could have fluctuation of accuracy. To minimize this weakness, both automated GPA program and MD slope were evaluated to assess progression of VF. Also, strict reliability assessment was performed when including subjects. Third, our study was limited because it could not evaluate the effect of systemic vasoactive medication such as BB for hypertension medication on VD. According to chart review, we identified that only two patients took systemic beta-blocker as hypertension medication, but if the patients were prescribed drugs at other hospitals, there must be missed information. A larger number of study participants might be needed for assessing the relationship between systemic vasoactive medication and VD. Fourth, we could not totally exclude the patients with cataract or multifocal intraocular lens insertion that could affect the quality of OCTA image. So, we tried to minimize the interference by restricting the collected OCTA with image quality over 70.

In conclusion, the status of the deep retinal vascular circulation might be a useful and direct indicator of local ocular blood circulation, and OCTA could be used to assess it. Indeed, deep VD values, as measured by OCTA, might be predictive of the risk of deterioration of visual function in glaucoma.

## Materials and Methods

### Subjects

This study was approved by the Institutional Review and Ethics Boards of Seoul St. Mary’s Hospital, South Korea. Informed consent was obtained from every subject. All work was performed according to the tenets of the Declaration of Helsinki. The subjects of the current study were enrolled from the ongoing Catholic Medical Center Glaucoma Progression Study (CMC-GPS), which was initiated in 2009 at Seoul St. Mary’s Hospital. The collected data in this study was accumulated from 2009 to 2018.

A total of 104 eyes from 104 OAG patients with IOP under 21 mmHg was included. Only patients with at least 5 years of follow-up were included in this study and all subjects visited our clinic every 3 months with fundus photography. VF test and OCT evaluation were performed at 6-month intervals for the first 3 years after diagnosis of glaucoma and annually thereafter.

The inclusion criteria for the study were as follows: (1) age between 20 and 79 years, (2) IOP ≤ 21 mmHg with IOP-lowering medication, (3) best corrected visual acuity of 20/40 or better, (4) open angle on gonioscopy, (5) spherical equivalent within ±5.0 diopters, and (6) no history of disease affecting visual pathways, retinal disease, or optic neuritis. If both eyes satisfied the inclusion criteria, the right eye was selected. Patients with a history of incisional glaucoma surgery or laser trabeculoplasty were excluded.

All subjects underwent comprehensive ophthalmic examinations, which included the following: report of systemic disease, best-corrected visual acuity, slit-lamp examination, Goldmann applanation tonometry, gonioscopy, and dilated stereoscopic examination of the optic disc. Red-free fundus photography (Canon, Tokyo, Japan), Cirrus OCT (Carl Zeiss Meditec), and Humphrey VF examination using the Swedish interactive threshold standard algorithm (SITA) 24-2 (Carl Zeiss Meditec) were also performed.

Glaucomatous VF defects satisfied following the following conditions: (1) glaucoma hemifield test results were outside normal limits (2) ≥3 adjacent points with a probability of <5% of the normal population, with one of these points having a probability of <1%, or (3) a pattern standard deviation (PSD) with a P value < 5%. Two glaucoma specialists confirmed the glaucomatous field defects (SJJ and HYP). We considered the VF results to be reliable as follows: when fixation loss was <20%, the false-positive rate was <15%, and the false-negative rate was <15%.

### Vessel density evaluation using OCT angiography

The vascular status of the macula was imaged using swept-source OCTA device (DRI OCT Triton; Topcon). The central wavelength of 1050 nm and scan speed of 100,000 A-scans per second were applied. A 3 × 3 mm macular scan was obtained and active eye tracker system was used to reduce motion artifacts during imaging. En face images via automated layer segmentation were acquired. The superficial retinal vascular plexus corresponds to the region starting from 2.6 μm below the internal limiting membrane to 15.6 μm below the junction of the inner plexiform layer (IPL) and INL (IPL/INL). The deep retinal vascular plexus reach from 15.6 μm below IPL/INL to 70.2 μm below IPL/INL.

To calculate macular VD, a binary slab image was created by ImageJ software (National Institutes of Health, Bethesda, MD, USA) as described by our group and others^11,37,38^. The binarized image was converted to a red-green-blue (RGB) color model and then split into three channels corresponding to red, green, and blue. The red channel was chosen as the reference. After using the “adjust threshold” tool, which automatically sets lower and upper threshold values, images were segmented into an area of interest and background. The white pixels were designated “vessel” and black pixels designated “background”, and the VD was calculated as a percentage from the total area of the white pixels divided by the total area of the image.

To reduce image quality fluctuation, the examinations were performed by one trained examiner. Only images with image quality scores greater than 70 were selected. OCTA images containing motion artifacts or blurred vessel contours that interfered with the clarity of vascular status were excluded. In the case of deep retinal angiogram images with projection artifacts from superficial retinal vessel, those images were also excluded. The quality of en face image was independently evaluated by three glaucoma specialists (SJJ, HYP, and CKP).

### Definition of VF progression

The presence of glaucomatous VF progression was assessed by trend-based analysis using Guided Progression Analysis (GPA) software. In trend-based analysis, eyes with a significant negative regression slope with a probability value less than 0.05 were defined as having glaucomatous VF progression. In addition, the rate of VF progression was calculated as the mean deviation (MD) slope and recorded as decibels per year (dB/yr). To minimize the effect of subjectivity in VF testing, all patients were required to produce at least 5 reliable VF tests, and the first result was excluded from the calculation of the MD slope.

### Statistical analysis

All descriptive results were calculated as the value of mean and standard deviation. All OCT parameters used for analysis were measurements from the initial examination. Correlation coefficients were calculated to evaluate the relationship between the MD slope and clinical or ocular parameters by Pearson correlation analysis. Linear regression analysis was applied to assess meaningful factors affecting the MD slope. After categorizing subjects as progressors or non-progressors according to GPA analysis, we used the Student’s t test and chi-square test to compare continuous or categorical variables between progressors and non-progressors. Logistic regression analysis was used to identify factors associated with glaucomatous VF progression, and the Cox proportional hazards model was applied to reflect different follow-up periods between subjects. All statistical analyses were performed with SPSS version 24.0 (SPSS Inc., Chicago, IL, USA) and *P* < 0.05 was considered to be statistically significant.

## Supplementary information

Supplementary table 1
