# Supplementary material for: Association of Retinal Blood Flow with Progression of Visual Field in Glaucoma
Source: Sci Rep. 2019 Nov 14;9:16813. doi: 10.1038/s41598-019-53354-4 (PMC6856104; doi:10.1038/s41598-019-53354-4)
Supplement: Supplementary file 1 — Supplementary table 1 [file 41598_2019_53354_MOESM1_ESM.docx]

**Association of Retinal Blood Flow with Progression of Visual Field in Glaucoma**

*Soo Ji Jeon, Da-Young Shin, Hae-Young Lopilly Park, Chan Kee Park*

Department of Ophthalmology, Seoul St. Mary’s Hospital, College of Medicine, The Catholic University of Korea, Seoul, Republic of Korea

**Corresponding author**

Chan Kee Park, MD, PhD

Department of Ophthalmology, Seoul St. Mary’s Hospital, College of Medicine, The Catholic University of Korea, 222 Banpo-daero, Seocho-gu, Seoul 06591, Republic of Korea

E-mail: ckpark@catholic.ac.kr

Tel : 82-2-2258-1188

Fax : 82-2-599-7405

**Key words** : optical coherence tomography angiography, open angle glaucoma, visual field, glaucoma progression

Supplementary table 1. Comparisons of VDs, MD, and IOP between different types of topical medications

| Topical prostaglandin | (+) | (-) | *P* value |
| --- | --- | --- | --- |
| Superficial VD, % | 25.73 (±3.75) | 26.11 (±3.73) | 0.632 |
| Deep VD, % | 31.08 (±1.97) | 31.76 (±2.36) | 0.130 |
| Initial MD, dB | -4.28 (±6.07) | -4.49 (±4.01) | 0.863 |
| Initial IOP, mmHg | 15.94 (±2.79) | 16.55 (±2.93) | 0.317 |
| Topical beta blocker | (+) | (-) | *P* value |
| Superficial VD, % | 24.96 (±3.97) | 26.81 (±3.22) | 0.011 |
| Deep VD, % | 31.34 (±2.26) | 31.25 (±1.96) | 0.821 |
| Initial MD, dB | -6.11 (±5.68) | -2.45 (±2.80) | 0.001 |
| Initial IOP, mmHg | 16.93 (±3.11) | 15.28 (±2.23) | 0.003 |

VD : vessel density; MD : mean deviation; IOP : intraocular pressure
